# Prevalence of Cryptococcal Antigenemia and Associated Factors among HIV/AIDS Patients at Felege-Hiwot Referral Hospital, Bahir Dar, Northwest Ethiopia

**DOI:** 10.1155/2021/8839238

**Published:** 2021-01-18

**Authors:** Mohabaw Jemal, Teshiwal Deress, Teshome Belachew, Yesuf Adem

**Affiliations:** ^1^University of Gondar, College of Medicine and Health Sciences, School of Biomedical and Laboratory Sciences, Department of Medical Microbiology, Gondar, Ethiopia; ^2^University of Gondar, College of Medicine and Health Sciences, School of Biomedical and Laboratory Sciences, Unit of Quality Assurance and Laboratory Management, Gondar, Ethiopia; ^3^Bahir Dar University, College of Medicine and Health Sciences, School of Health Sciences, Department of Medical Laboratory Science, Unit of Medical Microbiology, Bahir Dar, Ethiopia

## Abstract

**Background:**

Cryptococcosis is the most common opportunistic fungal infection. High morbidity and mortality are frequently observed among hospitalized HIV/AIDS patients, particularly having CD4 count ≤100 cells/*μ*l. Therefore, this study aimed to determine the prevalence of cryptococcal antigenemia and associated factors among HIV/AIDS patients.

**Methods:**

A hospital-based cross-sectional study was conducted among 140 HIV/AIDS patients. A cryptococcal antigen test was performed for all patients along with medical chart and laboratory registration book review. Cryptococcal antigen was detected from serum by using Remel Cryptococcal Antigen Test Kit. Data related to possible associated factors were extracted from patients' charts and laboratory registration book. Data were coded, entered, and analyzed using SPSS version 20. Logistic regression analysis was done to see the association between dependent and independent variables. A *P* value <0.05 was considered statistically significant. Finally, data were presented in the form of texts, figures, and tables.

**Result:**

Among 140 serum cryptococcal antigenemia-tested study subjects, 16 (11.43%) were positive for serum cryptococcal antigen. Of them, 43.8% (7/16) were pulmonary tuberculosis coinfected, 31.2% (5/16) were extrapulmonary tuberculosis positive, and 25% (4/16) had bacterial bloodstream infections. In addition, 68.7% (11/16) had CD4 count less than 100 cells/*μ*l, 18.7% (3/16) had CD4 count 100–150 cells/*μ*l, 50% (8/16) were antiretroviral therapy defaulters, and 31.3% (5/16) were naïve. In this study, the majority, 75% (12/16), of the serum cryptococcal antigen-positive subjects were clinical stage IV. Of the assessed associated factors, tuberculosis coinfection (AOR: 0.04; 95% CI [0.005–0.25]) and antiretroviral therapy status (AOR: 0.02; 95% CI [0.001–0.5]) were significantly associated factors enhancing serum cryptococcal antigenemia.

**Conclusion:**

In this study, the high rate of cryptococcal antigenemia was observed among hospitalized HIV/AIDS patients, and it is alarming and highlights the need for improving CD4 status, expanding serum cryptococcal antigen screening, and strengthening regular cryptococcal antigenemia surveillance systems.

## 1. Introduction

### 1.1. Background

Cryptococcosis is one of the most common opportunistic systemic fungal infections among human immune-deficiency virus/acquired immune-deficiency syndrome (HIV/AIDS) patients and other immune-suppressed individuals resulted from cancer chemotherapy, diabetes mellitus, sarcoidosis, steroid therapy, renal transplantation, or other genetically related diseases. *Cryptococcus neoformans and Cryptococcus gattii* are the most common causative agents of cryptococcosis [1].

Cryptococcosis is an important contributor to morbidity and mortality among HIV/AIDS patients [2,3]. Cryptococcus meningitis is an opportunistic fungal infection of the central nervous system (CNS). HIV is one of the most important factors for the development of cryptococcal infection [4]. Cryptococcal infections are the major cause of death among HIV/AIDS patients accounting for 20–25% of HIV/AIDS-related mortality in Africa [5]. Therefore, screening of serum cryptococcal antigenemia and early antifungal treatment are important for reducing deaths by cryptococcosis [6, 7]. Serum cryptococcal antigen is detectable in blood weeks to months before the development of clinical symptoms. This prolonged subclinical period of asymptomatic infection presents an opportunity to identify persons with asymptomatic or early treatment [8]. Insufficient antiretroviral treatment of HIV/AIDS patients can increase the rate of cryptococcosis infection among them [9].

Serum cryptococcal antigen-positive HIV/AIDS patients taking no ART treatment are at high risk of developing symptomatic cryptococcal meningitis [9, 10]. The majority of the deaths that occur among AIDS patients in Ethiopia are in the first four months of ART [11]. Different studies from different corners of the world showed an increased prevalence of cryptococcal infections. However, the mortality and morbidity rate of cryptococcosis and its associated factors among HIV/AIDS patients in Ethiopia are poorly documented. Therefore, this study aimed to screen serum cryptococcal antigen and its associated factors among HIV/AIDS patients which will help health policymakers in the development of strategies for early diagnosis and proper treatment of suspected patients.

## 2. Materials and Methods

### 2.1. Study Setting

This study was conducted at Felege-Hiwot Referral Hospital, Bahir Dar, Ethiopia. Bahir Dar city is located to the northwest and 540 km away from Addis Ababa, the capital city of Ethiopia. Felege-Hiwot Referral Hospital was purposefully selected with the fact that it is one of the biggest tertiary level referral hospitals in the region visited by around seven million people per year from the surrounding zones and nearby regions. Felege-Hiwot Referral Hospital has different departments including a diagnostic laboratory and around 400 beds, nine operating tables, one ART clinic with an ART laboratory, and one ART pharmacy.

### 2.2. Study Design and Period

A hospital-based cross-sectional study was conducted to determine serum cryptococcal antigen and associated factors among hospitalized HIV/AIDS patients from July 15, 2019, to December 15, 2019.

### 2.3. Study Subjects

All HIV/AIDS patients who were suspected of having cryptococcosis and having laboratory diagnosis for serum cryptococcal antigen during the study period were included as study subjects.

### 2.4. Inclusion Criteria

All HIV/AIDS patients who were suspected of having cryptococcosis during the study period were included.

### 2.5. Exclusion Criteria

Patients receiving antifungal treatment and patients less than 15 years were excluded from this study.

### 2.6. Sample Size

During the five months of the study period, a total of 140 cryptococcal infection-suspected HIV/AIDS patients visited Felege-Hiwot Referral Hospital and included in this study.

### 2.7. Data Collection and Laboratory Methods

A pretested, structured questionnaire was used to collect sociodemographic information. Data related to associated factors such as recent CD4 count, ART treatment, clinical stage, and current history of tuberculosis (pulmonary tuberculosis or extrapulmonary tuberculosis) were collected from patients' charts and laboratory registration books.

#### 2.7.1. Sample Collection and Laboratory Procedures

In the Felege-Hiwot Referral Hospital laboratory, different clinical specimens are being processed with respective standard procedures. For instance, during this study, screening of pulmonary and extrapulmonary tuberculosis was done by gene expert and fluorescent microscopy techniques, and clinical specimens like sputum, gastric lavage, and cerebrospinal fluid were used for diagnosis. Venous blood has been also used for the diagnosis of blood stream infections. Blood for screening serum cryptococcal antigen was collected under strict aseptic conditions using the venipuncture technique. Before blood collection, careful skin cleaning using 2% iodine tincture and 70% alcohol was done to prevent contamination. Serum samples were prepared through centrifugation and tested with Remel Cryptococcal Antigen Test Kit (Remel, London, UK). The serum was treated with protease enzyme, heated at 100°C for five minutes, cooled to room temperature, and then mixed with latex reagent. Clumping within 10 minutes of rotation was interpreted as the specimen is positive for cryptococcal antigen. Bloodstream infection of the study participants was screened by manual blood culture.

#### 2.7.2. Data Analysis and Interpretation

All the data were coded and entered into Epi data 3.1 and exported into SPSS 20 version statistical software for analysis. During analysis, cross-tabulations and odds ratios were used to compare frequencies. Descriptive statistics were computed to describe the study subjects in relation to relevant variables. Logistic regression analyses were done to determine factors associated with cryptococcal antigenemia. A variable with a *P* value <0.2 in bivariate logistic regression was included in multivariate analysis. Crude and adjusted odds ratios were calculated to quantify the strength of association between the rate of cryptococcal infection and associated factors. The 95% confidence interval was used, and associated factors with a *P* value <0.05 in multivariate analyses were considered statistically significant. Finally, data were presented in the form of texts, tables, and graphs.

### 2.8. Ethical Consideration

Ethical clearance was obtained from the Departmental Research and Ethics Review Committee (DRERC) of Addis Ababa University. Then, permission was obtained from the Felege-Hiwot Referral Hospital to access data from the study population. Written consent was obtained from all eligible subjects and informed about the purpose of the study and their willingness to take part in the study. All information got from the study participants was coded to maintain confidentiality.

## 3. Results

### 3.1. Sociodemographic Characteristics

A total of 140 HIV/AIDS hospital-admitted patients were enrolled in this study, of whom 59 (42%) and 81 (58%) were males and females, respectively. All study subjects were in the age range of 15–60 years (mean ± SD = 35 ± 11.8 years). 62 (44%) of them were married, 100 (71.4%) were literates, and over 70% of them came from urban areas (Table 1).

### 3.2. Prevalence of Cryptococcal Antigenemia and Coinfections

In the current study, the prevalence of cryptococcal antigenemia was 11.43% (16). Of this, 43.8% (7/16) were pulmonary tuberculosis positive, 31.2% (5/16) were extrapulmonary tuberculosis positive, and 25% (4/16) had bacterial bloodstream infections. The majority (68.7%) of serum cryptococcal antigen-positive participants had CD4 count less than 100 cells/*μ*l, and they were ART defaulters (Table 1).

### 3.3. Factors Associated with Cryptococcal Antigenemia

Of the assessed associated factors, tuberculosis coinfection (AOR: 0.04; 95% CI [0.005–0.25]) and antiretroviral therapy status (AOR: 0.02; 95% CI [0.001–0.5]) were significantly associated factors enhancing serum cryptococcal antigenemia (Table 2).

**Figure 1:**
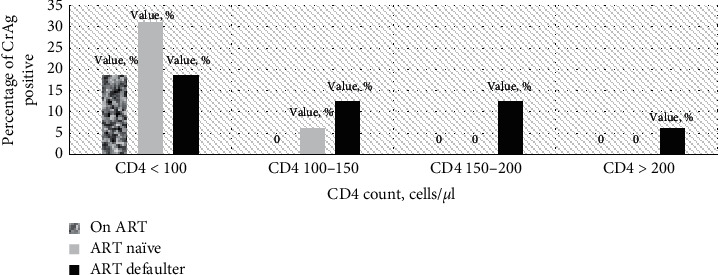
Percentage of cryptococcal antigen positive HIV/AIDS patients associated with CD4 count and ART use.

In the current study, the prevalence of cryptococcal antigenemia was more frequently detected among ART defaulters, 50% (8/16). More than 31% of the cryptococcal antigen-positive participants had CD4 count less than 100 cells/*μ*l, and they were on ART (Figure 1).

## 4. Discussion

HIV-infected people are at high risk of developing different opportunistic infections, including fungal and bacterial infections. Late presentations to care of HIV-infected people will put them at risk for the development of the cryptococcal disease. In resource-constrained settings, continuous surveillance of the cryptococcal infection in HIV‐infected people and associated factors is quite limited because of lack of cheap diagnostic tools, but it is important to support the national treatment program. For these resource-constrained countries, Remel Cryptococcal Antigen Test Kit might be a good possible option for diagnosing cryptococcal antigenemia among HIV/AIDS patients because according to the manufacture's instruction, this test kit has 100 percent specificity and sensitivity. In the current study, the prevalence of cryptococcal antigenemia was 11.43%. However, this percentage was significantly lower than previous reports in India (33.33%) and in China (21%) [12]. Another similar study was conducted among hospitalized AIDS patients on the continent level, and 69%, 80%, and 80% cryptococcal antigenemia was reported in Africa, Europe, and the USA, respectively [12]. This magnitude was significantly higher than the study done in Ethiopia among HIV/AIDS patients receiving antiretroviral therapy, which is 8.8% and 10.2% [8, 13].

In this study, cryptococcal antigenemia was more prevalent among females (56%) than male patients (44%). This percentage was almost similar to previous reports from Addis Ababa, Ethiopia (47% females and 52.9% males) [14]. The difference in prevalence between females and males might be resulted due to the difference of exposure rather than the difference in host susceptibility and environmental exposure, hormonal, and genetic predisposition [15–17]. Over 69% of the serum CrAg-positive participants were in the age group of 25–44 years. Similarly, different studies from many parts of the world showed that the prevalence of cryptococcosis among HIV-infected patients was higher in a similar age group [18, 19].

The screening of serum cryptococcal antigen was also done among HIV/AIDS patients having tuberculosis coinfection. In this study, the majority of the serum CrAg-positive individuals had a clinical history of tuberculosis. Cryptococcal antigenemia positivity rate was higher among patients with a history of both pulmonary (7/16 (43.8%) *P*=0.001) and extrapulmonary tuberculosis coinfection (5/16 (31.2%), *P*=0.006) compared to patients with other blood stream infections, 4/16 (25%). In this study, the association between cryptococcal antigenemia positivity rate and antiretroviral therapy status was also statistically significant (AOR: 0.02; 95% CI [0.001–0.5]). Our result was consistent with a result reported in another Ethiopian study [8]. Therefore, antiretroviral therapy with good adherence is an important consideration for AIDS patients for reducing the prevalence of cryptococcal antigenemia [8, 10, 20].

### 4.1. Limitation

We, the authors, could not perform other laboratory procedures like fungal blood culture, chest radiography, and determination of liver enzyme levels because of the resource limitation.

## 5. Conclusions

In this study, we found a previously unreported high prevalence of serum cryptococcal antigen among HIV/AIDS patients. Particularly, it was quite high among patients having CD4 counts less than 100 cells/*μ*l, and it is alarming and highlights the need for improving CD4 status, expanding serum cryptococcal antigen screening, and strengthening regular cryptococcal antigenemia surveillance systems.

## Figures and Tables

**Table 1:** Sociodemographic characteristics of HIV/AIDS patients screened for serum cryptococcal antigen in Felege-Hiwot referral hospital, Bahir Dar, Ethiopia.

Characteristics	Pos., *N* (%)	Neg., *N* (%)	Total, *N* (%)
Sex			
Male	7 (44)	52 (42)	59 (42)
Female	9 (56)	72 (58)	81 (58)

Age			
15–24	3 (19)	16 (13)	19 (14)
25–44	11 (69)	76 (63)	87 (63.5)
>45	2 (12)	29 (24)	31 (22.6)

Residence			
Urban	9 (56)	91 (73.4)	100 (71)
Rural	7 (44)	33 (26.6)	40 (29)

Marital status			
Unmarried	6 (37.5)	33 (26.6)	39 (28)
Married	5 (31.2)	57 (46)	62 (44)
Others^a∗^	5 (31.2)	34 (27.4)	39 (28)

Educational level			
Illiterate	4 (25)	36 (29)	40 (28.6)
Literate	12 (75)	88 (71)	100 (71.4)

Occupation			
Student	1 (6.2)	17 (14)	18 (12.8)
House wife	2 (12.5)	20 (16)	22 (15.7)
Employed	11 (68.8)	71 (57)	82 (58.6)
Others	2 (12.5)	16 (13)	18 (12.8)

CD4+ (cells/ml)			
<100	11 (68.7)	7 (5.6)	18 (12.9)
100–150	3 (18.7)	31 (25)	34 (24.3)
150–200	1 (6.3)	14 (11.3)	15 (10.7)
>200	1 (6.3)	72 (57.2)	73 (52.1)

Antiretroviral therapy status			
On ART	3 (18.8)	67 (54)	70 (50)
ART naïve	5 (31.2)	38 (30.6)	43 (30.7)
ART defaulter	8 (50)	19 (15.3)	27 (19.3)

Bacterial infection status (*n* = 79)			
Pulmonary TB	7 (43.8)	7 (11.1)	14 (17.7)
Ex pulmonary TB	5 (31.2)	6 (9.5)	11 (13.9)
BSI	4 (25)	50 (79.4)	54 (68.4)

WHO clinical stage			
Stages I and II	1 (6)	105 (84.7)	106 (75.7)
Stage III	3 (19)	17 (13.7)	20 (14.3)
Stage IV	12 (75)	2 (1.6)	14 (10)

*Note.* WHO, World Health Organization; others, widow and divorced; BSI, bacterial infection isolated by blood culture; employed, government and self-employed; others, jobless and children.

**Table 2:** Bivariate and multivariate analysis of factors associated with serum cryptococcal antigenemia among HIV/AIDS patients in Felege-Hiwot Referral Hospital, Bahir Dar, Ethiopia.

Variable	Pos., *N* (%)	Neg., *N* (%)	*P* value	COR (95% CI)	*P* value	AOR (95% CI)
Sex						
Male	7 (44)	52 (42)	1		1	
Female	9 (56)	72 (58)	0.890	1 (0.377–3)	0.329	0.4 (0.05–2.7)

Age						
15–24	3 (19)	16 (13)	0.300	0.4 (0.056–2.4)	0.268	0.2 (0.007–3.9)
25–44	11 (69)	76 (63)	0.354	0.5 (0.1–2.3)	0.586	0.6 (0.07–4.6)
>45	2 (12)	29 (24)	1			

Marital status						
Unmarried	6 (37.5)	33 (26.6)	0.745	0.8 (0.23–2.9)	0.637	1.7 (0.2–13)
Married	5 (31.2)	57 (46)	0.440	1.7 (0.45–6.2)	0.846	0.8 (0.1–6.3)
Others	5 (31.2)	34 (27.4)	1			

Educational status						
Illiterate	4 (25)	36 (29)	1	1		
Literate	12 (75)	88 (71)	0.737	0.82 (025–2.7)	0.366	0.4 (0.04–3.2)

Occupation						
Student	1 (6.2)	17 (14)	1		1	
House wife	2 (12.5)	20 (16)	0.676	0.6 (0.05–7)	0.580	0.4 (0.012–12)
Employed	11 (68.8)	71 (57)	0.369	0.4 (0.05–3)	0.185	0.14 (0.008–2.6)
Jobless	2 (12.5)	16 (13)	0.554	0.5 (0.04–5.7)	0.340	0.19 (0.006–5.9)

Residence						
Urban	9 (56)	91 (73.4)	0.160	2 (0.74–6)	0.831	1.3 (0.2–10)
Rural	7 (44)	33 (26.6)	1			

Bacterial status (*n* = 79)						
Pulmonary	7 (43.8)	7 (11.1)	0.001	0.08 (0.02–0.35)	0.001	0.04 (0.005–0.25)
Ex pulmonary	5 (31.2)	6 (9.5)	0.003	0.1 (0.02–0.46)	0.006	0.07 (0.01–0.47)
BSI	4 (25)	50 (79.4)	1			

CD4 status						
<100	11 (68.7)	7 (5.6)	0.007	0.05 (0.005–0.4)	0.108	0.04 (0.001–2)
100–150	3 (18.7)	31 (25)	0.800	0.74 (0.07–7.7)	0.343	4.6 (0.2–110)
151–200	1 (6.3)	14 (11.3)	1			
>200	1 (6.3)	72 (59.4)	0.257	5 (0.3–87)	0.096	31 (0.54–1865)

ART status						
On ART	3 (18.8)	67 (54)	1			
ART naïve	5 (31.2)	38 (30.6)	0.155	0.34 (0.07–1.5)	0.161	0.09 (0.004–2.5)
ART defaulter	8 (50)	19 (15.3)	0.002	0.1 (0.026–0.44)	0.019	0.02 (0.001–0.5)

*Note*. 1 = as a reference; others, widow and divorced; BSI, bacterial infection isolated by blood culture; AOR, adjusted odds ratio; OR, crude odds ratio; CI, confidence interval.

## Data Availability

Data and supporting material associated with this study will be shared upon request.
